# Detection of Tert-Butylhydroquinone in Edible Oils Using an Electrochemical Sensor Based on a Nickel-Aluminum Layered Double Hydroxide@Carbon Spheres-Derived Carbon Composite

**DOI:** 10.3390/foods13213431

**Published:** 2024-10-28

**Authors:** Jin Zhang, Jingrong Chen, Jiejun Li, Yixi Xie

**Affiliations:** 1 School of Materials and Chemical Engineering, Hunan City University, Yiyang 413000, China; tccdzdc@163.com; 2 Key Laboratory of Low Carbon and Environmental Functional Materials of College of Hunan Province, Yiyang 413000, China; 3 Hunan Provincial University Key Laboratory for Environmental and Ecological Health, Xiangtan University, Xiangtan 411105, China

**Keywords:** tert-butylhydroquinone, nickel-aluminum double hydroxide, electrochemical sensor, food additive testing

## Abstract

Phenolic antioxidants such as tert-butylhydroquinone (TBHQ) can prolong the shelf life of edible oils by delaying the oxidation process. The excessive use of TBHQ can damage food quality and public health, so it is necessary to develop an efficient TBHQ detection technique. In this work, nickel-aluminum double hydroxide (NiAl-LDH) was grown on glucose carbon spheres (GC), which formed porous carbon nanomaterials (named NiAl-LDH@GC-800) after pyrolysis at 800 °C. The successful synthesis of the material was verified by scanning electron microscopy, X-ray diffraction, and X-ray photoelectron spectroscopy. The obtained NiAl-LDH@GC-800 was dopped onto a glass carbon electrode to prepare an electrochemical sensor for TBHQ. The synergistic effect of porous carbon and Ni metal reduced from NiAl-LDH by high-temperature calcination accelerated the electron transfer rate and improved the sensitivity of the sensor. The prepared sensor showed a low limit of detection (LOD) of 8.2 nM, a high sensitivity (4.2 A·M^−1^), and a good linear range (20~300 µM) in detecting TBHQ. The sensor was also successfully used for TBHQ detection in edible oils, including chili oil, peanut oil, and rapeseed oil.

## 1. Introduction

To slow down the oxidation process of cooking oil for long-term preservation, manufacturers will add appropriate amounts of oil-soluble antioxidants, such as tert-butylhydroquinone (TBHQ), butylhydroxyanisole, dibutylhydroxytoluene, and other food additives to the oil at a later stage of processing [1,2]. TBHQ has become one of the widely used food additives in oil due to its strong antioxidant ability, odorless nature, good stability, and low cost [3,4]. However, the use of TBHQ in high doses can negatively affect food quality and public health, so many countries have established safety restrictions on the use of TBHQ [5]. The use of TBHQ in food has been banned in countries and regions such as the European Union, Japan, Canada, Hong Kong, etc. [6]. The Food and Drug Administration (FDA, USA) stipulate that the use of TBHQ in food must be controlled within 200 μg/g [7]. Therefore, it is very important to quantify TBHQ in edible oils efficiently. Various methods have been developed for TBHQ detection, including high-performance liquid chromatography (HPLC) [8], gas chromatography [9], and fluorescence sensing [10]. These methods usually suffer from the drawbacks of time-consuming sample pre-treatment, complicated operation, the use of toxic solvents, and expensive instruments. In contrast, an electrochemical sensor with high sensitivity and stability provides researchers with a faster and simpler alternative [11,12].

Li et al. electrodeposited Au nanoparticles on molybdenum disulfide-graphene to prepare a sensor that could detect both TBHQ and butylated hydroxyanisole in food products, due to the catalytic properties of Au nano and the heterogeneous structure of molybdenum disulfide-graphene [13]. Monteiro et al. developed a photoelectrochemical sensor using CdSe/ZnS core-shell quantum dots and lithium tetracyanoethylate [14]. The incorporation of the quantum dots and lithium tetracyanoethylate resulted in a significant reduction in the charge transfer resistance of the composite material, with large response currents in the detection of TBHQ in foods [14]. Jenisha et al. synthesized hierarchically structured antimony tungstate nanoflowers in combination with acidified carbon nanotubes. The composites showed good electrocatalytic ability and were successfully used for the detection of TBHQ in milk [6]. Yue et al. constructed a molecularly imprinted electrochemical sensor using PdAuNPs/ERGO nanomaterials [15]. Highly catalytically active Pd-Au bimetallic nanoparticles and reduced graphene oxide synergistically amplified the electrochemical signals, and molecular imprinting efficiently and selectively recognized TBHQ [15]. Many materials were successfully employed to detect TBHQ, but most of the raw materials they used were expensive, which increased the cost of making the sensors. Therefore, the development of low-cost, efficient, and sensitive TBHQ is necessary.

Layered double hydroxides (LDH) can be prepared and synthesized from low-cost precursors by various synthetic routes and are widely used by researchers [16,17]. Recent developments in LDH nanomaterials are of great significance in advancing sensor technology for food detection [18,19]. Fu et al. used nickel-cobalt layered double hydroxides (Ni-Co LDH) fabricated by electrostatic spinning and chemical deposition techniques and developed a sensor with excellent performance for glucose [20]. Zhu et al. prepared ultrathin CoAl layered double hydroxide nanosheets by the co-precipitation method to construct a QCM humidity sensor [21], which can monitor the humidity in vegetable packages. Cui et al. used a calcined Cu-LDH with a gold nanoparticles composite sensor which detects nitrate in ham [22]. It was found that LDHs can be used as catalysts with good hydrolytic and chemical stability [23,24]. The use of LDHs improves the selectivity and sensitivity of electrochemical sensors [25]. The advantages of the low cost and excellent catalytic properties of hydrotalcite hold great promise for applications in food analysis [25,26]. However, hydrotalcite material alone has the disadvantage of poor electrical conductivity, and the addition of a carbon source to the material is a good solution. Recent advances in the design and synthesis of carbon materials, especially nanomaterials, have demonstrated powerful electrochemical sensing detection capabilities [27,28]. Yang et al. designed a porous carbon (Co-N-C@PC) composite electrochemical sensor for the detection of acetaminophen, in which the porous carbon was derived from sodium citrate [29]. Pu et al. pyrolytically synthesized oak biomass charcoal (BC) and BC-Au composites for the simultaneous detection of Cd^2+^, Pb^2+^, and Hg^2+^ [30]. Tran et al. developed a biosensor for detecting breast cancer cells in serum, and the carbon source of the material was derived from passion fruit juice [31]. Carbon materials provide a large surface area and active sites for adsorption, thus increasing the sensitivity of analyte detection [32,33]. In addition, they can be used as supports for other modified materials such as metals [34], metal oxides [35], metal hydroxides [36,37], and conducting polymers [30,37]. The sources of carbon in materials are varied and the right source of carbon can improve the performance of the sensor.

Based on the above studies, NiAl layered double hydroxide with low-cost raw materials and common glucose became our material for designing an efficient TBHQ electrochemical sensor. In our work, NiAl layered double hydroxides were synthesized on glucose carbon spheres (GC) and then pyrolyzed in a nitrogen atmosphere to prepare a porous carbon nanomaterial (named NiAl-LDH@GC-800) to construct an electrochemical sensor for TBHQ. The incorporation of GC reduces the buildup of NiAl-LDH growth, while the presence of NiAl-LDH during pyrolysis leads to the formation of a porous structure in the carbon spheres. The porous carbon obtained after the pyrolysis of GC at 800 °C gives the composites a larger specific surface area and more active sites and improves the electrical conductivity of the composites. We applied the synthesized porous carbon nanomaterials to the electrochemical sensing detection of TBHQ, and the sensor was responsive and showed a very good linear range compared to existing methods. In addition, the sensor was successfully used to identify and detect TBHQ in three daily cooking oils, demonstrating the practical application capability of the material.

## 2. Materials and Methods

### 2.1. Experimental Material

Nickel chloride (NiCl_2_·6H_2_O) and aluminum chloride (AlCl_3_·6H_2_O), tert-butylhydroquinone (TBHQ), urea, ammonium fluoride, glucose (Glu), Nafion reagent, Na_2_SO_4_, Mg (NO_3_)_2_, CaCl_2_, ascorbic acid (AA), salicylic acid (SA), citric acid (CA), resorcinol (RDP), o-nitrophenol (2-NP), and p-nitrophenol (4-NP) were obtained from Aladdin. Phosphate buffer solution (PBS) was prepared with NaCl, Na_2_HPO_4_, and NaHPO_4_, and the volume of PBS used for each test was 20 mL. All drugs and reagents were of analytical grade standard and could be used without further purification. Ultrapure water (18.25 MΩ cm) was used throughout the experiments.

### 2.2. Experimental Instruments

Scanning electron microscopy (SEM, H7700, Hitachi High-Technologies Corporation, Tokyo, Japan) and transmission electron microscopy (HRTEM, JEM-2100, Nippon Electronics Corporation, Tokyo, Japan) were used to characterize the morphology of NiAl-LDH@GC-800 and other materials. X-ray diffraction (XRD, Bruker D8 Discover, Karlsruhe, Germany) was used to characterize the crystal structure of the materials. The elemental composition of the material surface and its chemical state were analyzed using an X-ray photoelectron spectrometer (ESCALAB 250Xi, Thermo Fisher Scientific, Waltham, MA, USA). The materials were synthesized using an electric blast chamber (DHG9030A, Shanghai Jinghong Co., Ltd., Shanghai, China) and a tube muffle furnace (GMF-12-250, Shanghai Muffle Furnace Co., Ltd., Shanghai, China). A vacuum drying oven (VOS-30A, Kishan Instruments Co., Shanghai, China) was used to remove moisture from the materials. UV-Vis spectroscopy (UV-2450, Shimadzu, Kyoto, Japan) was used for the quantitative analysis of the actual samples. Electrochemical tests were performed using the electrochemical workstation (MULTI AUTOLAB M204, Metrohm, Herisau, Switzerland). The type of glassy carbon electrode was as follows (GCE): diameter = 3 mm, surface area = 7.068 mm^2^, and the manufacturer is Wuhan GaossUnion (Wuhan, China). Platinum wire was used as an auxiliary electrode, and an Ag/AgCl electrode was used as a reference electrode.

### 2.3. Parameters of Electrochemical Experiments

Electrochemical tests were performed in 0.1 M PBS using cyclic voltammetry (CV), differential pulse voltammetry (DPV), and electrochemical impedance spectroscopy (EIS). CV parameter setting: Start E (V) = −0.3, Stop E (V) = 0.3, Scan rate (V⋅s^−1^) = 0.1 (in PBS); Start E (V) = −0.2, Stop E (V) = 0.6, in 5.0 mM [Fe(CN)_6_]^3−/4−^ electrolyte-containing 0.1 M KCl (PFS). DPV parameter setting: Start E (V) = −0.3, Stop E (V) = 0.3, Step potential (V) = 0.005, Modulation amplitude = 0.025 V, Modulation time = 0.05 s, Scan rate (V⋅s^−1^) = 0.1 (in PBS). EIS parameter setting: Amplitude = 10 mV; pulse width = 5 mV; frequency range: 100 khz–0.1 Hz; open-circuit potential: 0.241 V. All electrolyte solutions (0.1 M PBS) were purged with nitrogen for 10 min in advance, and the nitrogen atmosphere was maintained throughout the experiment.

### 2.4. Synthesis Process

Synthesis of GC [38]: 2 g of glucose was dissolved in 60 mL of ultrapure water and stirred for 20 min; then, the solution was transferred to a 100 mL Teflon hot press vessel, heated at 180 °C for 4 h, cooled naturally, washed three times with ultrapure water, and dried under vacuum at 60 °C for 10 h. The black powder product is recorded as GC.

Synthesis of NiAl-LDH@GC: 50 mg of GC, 0.3 mmol of NiCl_2_·6 H_2_O, and 0.1 mmol of AlCl_3_·6 H_2_O were added into a mixture of 30 mL of ultrapure water and 30 mL of ethanol and stirred at room temperature for 1 h. Then, 2 mmol of urea and 2 mmol of NH_4_F were added and stirred for 20 min, and then the mixture was transferred to a Teflon-lined autoclave vessel and the reaction was heated at 160 °C for 6 h. After natural cooling, the precipitate was washed three times with ultrapure water and anhydrous ethanol until the pH was neutral to obtain a brown precipitate. The brown product was dried at 60 °C for 10 h under vacuum. The product obtained was noted as NiAl-LDH@GC. NiAl-LDH was synthesized without the addition of GC [39].

Synthesis of NiAl-LDH@GC-800: 30 mg of NiAl-LDH@GC was heated in a muffle furnace at T = 800 °C for 2 h at a ramp rate of 5 °C/min under 0.2 L/min of N_2_. After natural cooling, the black powder material was noted as NiAl-LDH@GC-800. NiAl-LDH@GC-700 and NiAl-LDH@GC-900 were prepared using the same method at temperatures of 700 °C and 900 °C, respectively. In addition, GC-800 and NiAl-LDH-800 were calcined.

### 2.5. Preparation of NiAl-LDH@GC-800/GCE

**Scheme 1:**
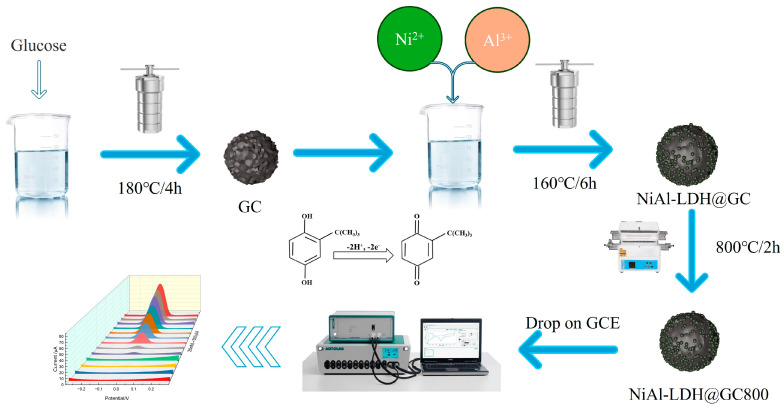
Schematic illustration of the synthesis process of NiAl-LDH@GC-800 and the fabrication of NiAl-LDH@GC-800/GCE.

First, an appropriate amount of 0.05 µm Al_2_O_3_ powder dissolved in ultrapure water was placed on a polishing cloth to polish the glassy carbon electrode (GCE) and sequentially sonicated with anhydrous ethanol and water for 5 min. Afterward, the glassy carbon electrodes were dried under an infrared lamp for about 3 min to be used for drop-coating the material. Then, 2 mg of NiAl-LDH@GC-800 was prepared as a suspension of 2 mg/mL in deionized water and uniformly sonicated for 2 h. In total, 6 μL of NiAl-LDH@GC-800 was pipetted onto the pre-treated glassy carbon electrode, and the electrode was dried by exposing it to the infrared light. After film formation, 3 μL of Nafion was taken by a pipette gun and dropped onto the treated GCE, which was then exposed to IR light for drying. In addition, we prepared other contrasting material electrodes using the same method. The synthesis process of NiAl-LDH@GC-800 and the fabrication of NiAl-LDH@GC-800/GCE are shown in Scheme 1.

### 2.6. Preparation of Test Samples

Edible chili oil, peanut oil, and rapeseed oil were purchased from local supermarkets. To determine the TBHQ content in various real samples, the real samples were treated as follows [40]: 0.5 mL of chili oil, peanut oil, and rapeseed oil were taken into centrifuge tubes and 5 mL of ethanol was added. A vortex shaker (JOANLAB, VM-300, Ningbo, China) was used to shake the samples in the three centrifuge tubes for 20 min to mix the samples thoroughly. In total, 2 mL of the supernatant was taken as the test sample.

## 3. Results and Discussion

### 3.1. Materials Characterization

#### 3.1.1. SEM Characterization

**Figure 1:**
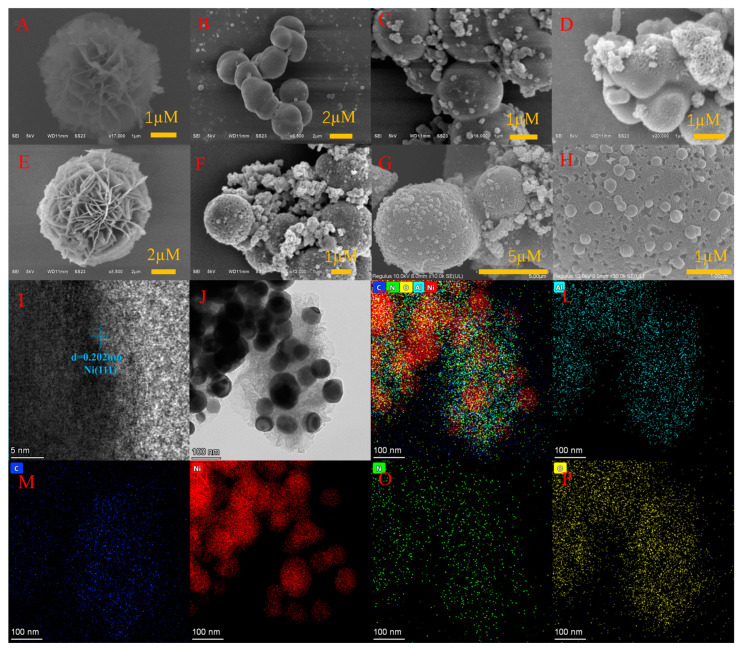
(**A**) SEM images of NiAl-LDH, (**B**) SEM images of GC, (**C**,**D**) SEM images of NiAl-LDH@GC, (**E**) SEM image of NiAl-LDH800, (**F**–**H**) SEM images of NiAl-LDH@GC800, (**I**) TEM images of NiAl-LDH@GC800, and (**J**–**P**) EDS element mapping of NiAl-LDH@GC800.

The surface morphology of the materials under different synthesis steps was determined using SEM. The nickel aluminum double hydroxide (NiAl-LDH) synthesized under hydrothermal conditions presents a petal-like shape consisting of nanosheets, as shown in Figure 1A. Glucose was hydrothermally hydrolyzed into carbon spheres (GC) at 180 °C, as shown in Figure 1B. As seen in Figure 1C, the GC surface acted as a nucleation substrate for Ni and Al ions under hydrothermal conditions in the presence of NH_4_F and urea, which hydrothermally formed granular double hydroxides on the surface of the carbon spheres, and in Figure 1D, it was found that petal-like double hydroxides were also formed on the surface of some of the carbon spheres, which verified that we had successfully grown the NiAl-LDH on the carbon spheres. After calcination at 800 °C for 2 h under the nitrogen atmosphere, the GC calcined a porous structure with some surface-loaded, relatively small double hydroxide particles embedded into the pores (Figure 1F–H). The high-resolution TEM image clearly shows the lattice surface of the Ni metal blob material on the surface of the carbon spheres, which was calculated and analyzed to be d = 0.202 nm, which coincides with (111) of the metal Ni annotation card (JCPDS card no. 04-0850) (Figure 1I). The distribution of various elements is observed through the EDS map of transmission electron microscopy (Figure 1G–J). It can be found that many small spheres are distributed on the surface of the carbon spheres (Figure 1J), and the elements C, Ni, Al, N, and O are uniformly distributed in the material (Figure 1G–K), where the Ni element is more obviously concentrated in the GC (Figure 1N).

#### 3.1.2. XRD and XPS Characterization

**Figure 2:**
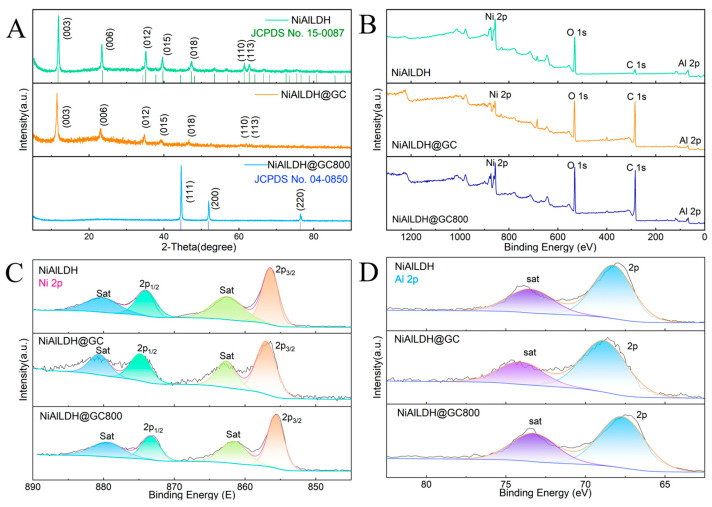
(**A**) XRD patterns of NiAl-LDH, NiAl-LDH@GC, and NiAl-LDH@GC800. (**B**) Overall spectrum for the NiAl-LDH, NiAl-LDH@GC, and NiAl-LDH@GC800. (**C**,**D**) XPS spectra of the Ni 2p, Al 2p.

The XRD patterns of synthesized NiAl-LDH and NiAl-LDH@GC are shown in Figure 2A, which are in good agreement with the structure of hydrotalcite described in previously reported literature [30]. The diffraction peaks observed at 11.13°, 22.89°, 39.17°, 46.33°, 60.77°, and 62.11° can be localized in the (003), (006), (012), (015), (018), (110), and (113) (JCPDS card no. 15-0087) planes, suggesting that NiAl-LDH on the GC spherical surface was successfully grown. Three diffraction peaks can be observed in the XRD pattern of NiAl-LDH@GC800 at 44.50°, 51.84°, and 76.37°, which is consistent with (111), (200), and (220) of (JCPDS card No. 04-0850), indicating that the pyrolysis material of Ni-based carbon has been successfully prepared. The fine shifts may be the effect of the little Al remaining after calcination at 800 °C.

Information on the elemental composition, chemical state, and molecular structure of NiAl-LDH, NiAl-LDH@GC, and NiAl-LDH@GC800 was analyzed by XPS. Figure 2B shows the XPS profiles of the three materials at 0–1300 eV binding energy, where the signals of nickel, aluminum, carbon, and oxygen can be seen. In the Ni 2p high-resolution XPS spectrum (Figure 2C), it is divided into two spin-orbit bimodal peaks, Ni 2p1/2 and Ni 2p3/2, and two satellite peaks [41]. The two distinct peak positions of Ni 2p3/2 and Ni 2p1/2 correspond sequentially to binding energies of 855.8 eV and 873.7 eV. The positions of their two satellite peaks are located at binding energies of 862.7 eV and 880.7 eV, respectively [42]. The presence of the satellite peaks suggests that the high-spin Ni^2+^ state exists in the preparation. Two peaks with binding energies of 74.08 eV and 68.81 eV, corresponding to Al^3+^ and satellite peaks, respectively, can be seen in Figure 2D [43], indicating the presence of Al^3+^ as Al(OH) in the NiAl-LDH material. The respective XPS characterization of the above three materials verified our successful growth of NiAl-LDH on the GC surface and the further successful calcination of the material.

#### 3.1.3. FTIR Analysis

Supplementary Figure S1A shows the FTIR of the four materials. The broad band around 3550 cm^−1^ is the -OH group of the water molecule. The characteristic peaks at 1372 cm^−1^ and 1048 cm^−1^ are the vibrational bands of carbonate ions present in the NiAl-LDH material [44]. The characteristic peak at 1637 cm^−1^ is the interlayer water’s OH group. The band around 673 cm^−1^ is the characteristic peak of Ni-O and Al-O vibration in LDH [45]. The FTIR spectra of NiAl-LDH@GC material still exist for GC material at 2922 cm^−1^, 1700 cm^−1^, 1383 cm^−1^, 1021 cm^−1^, 802 cm^−1^, and four distinct characteristic peaks of NiAl-LDH material. To some extent, this proved that NiAl-LDH was successfully grown in situ on the GC surface. The Zeta potential of each material was tested using a Zeta potential meter (Figure S1B). The Zeta potential of the glucose carbon spheres was −28.3 mV, the Zeta potential of the NiAl-LDH material was positive (Z_NiAl−LDH_ = 22.2 mV), and the Zeta potential of the GC material loaded with NiAl-LDH was −15 mV, and calcination made the material’s Zeta potential negative, which, side by side, confirms the successful loading of the target material.

### 3.2. Comparative Electrochemical Performance Analysis of Different Modified Electrodes

**Figure 3:**
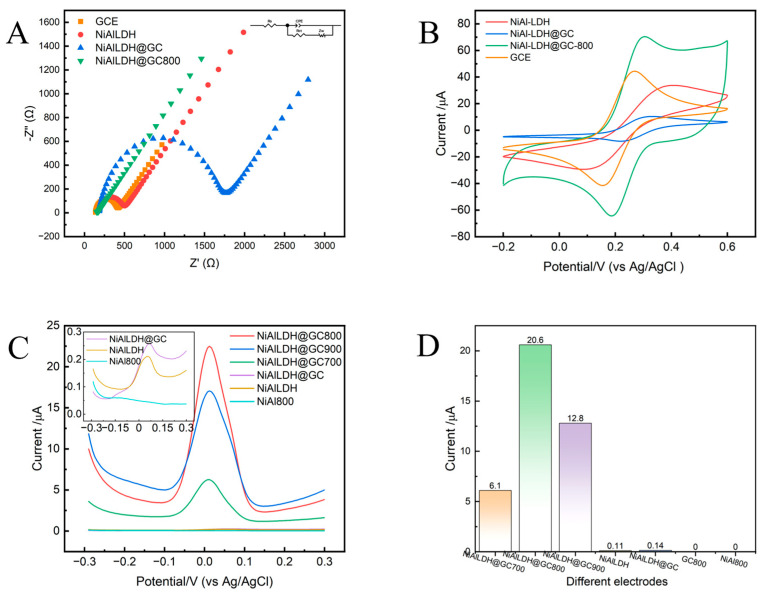
(**A**) The Nyquist plots of four modified electrodes. (**B**) CV of four modified electrodes in 5 mM [Fe(CN)_6_]^3−/4−^. (**C**) DPVs of NiAl-LDH, NiAl-LDH@GC, NiAl-LDH800, GC800, NiAl-LDH@GC700, NiAl-LDH@GC800, and NiAl-LDH@GC900 in 0.1 M PBS (pH = 7) containing 5 × 10^−6^ M TBHQ. (**D**) I_pa_ of NiAl-LDH800 and six contrasting material-modified electrodes.

Electrochemical impedance spectroscopy (EIS) is used to obtain impedance spectra by applying a small-amplitude sinusoidal potential (or current) perturbation signal to an electrochemical system and measuring the corresponding current (or potential) response generated by the system [46,47]. In electrochemical impedance testing, the equivalent circuit consists of a series resistor (Rs), a constant phase angle element (CPE), a charge transfer resistor (Rct), and a Warburg impedance (Zw). The corresponding Rct values were fitted through an equivalent circuit. In Figure 3A, the Nyquist curves of GCE, NiAl-LDH, and NiAl-LDH@GC show a clear semicircle at high frequency, indicating a higher resistance to electron transport on the surfaces of GCE, NiAl-LDH, and NiAl-LDH@GC. NiAl-LDH@GC800 has almost no semicircle in the high-frequency region, implying that it has a lower Rct value and a faster rate of electron transport. This may be due to the carbonization of the glucose carbon spheres during the high-temperature calcination process, which resulted in a significant increase in the electrical conductivity of the material. The above results indicate that NiAl-LDH@GC800 is an ideal material for the modification of the electrode with good electron transfer kinetics and a lower electron energy barrier.

Further, the redox qualities of each electrode surface were evaluated using the CV method in a 5 mM [Fe(CN)_6_]^3−/4−^ probe containing 0.1 M KCI [14]. As shown in Figure 3B, GCE, NiAl-LDH, NiAl-LDH@GC, and NiAl-LDH@GC800 exhibited a pair of redox peaks. Detailed CV data for the modified electrodes are presented in Table S1. Comparing the oxidation peak current (i_pa_) and the potential difference (∆Ep) between the redox peaks, GCE (∆Ep = 0.125 V, i_pa_ = 58.71 µA), NiAl-LDH (∆Ep = 0.281 V, i_pa_ = 25.63 µA), NiAl-LDH@GC (∆Ep = 0.127 V, i_pa_ = 12.51 µA), and NiAl-LDH@GC800 (∆Ep = 0.113 V, i_pa_ = 76.73 µA), we found that the redox peaks of NiAl-LDH and NiAl-LDH@GC are smaller than that of GCE, while NiAl-LDH@GC800 has the largest i_pa_ and the smallest ∆Ep. This result indicates that NiAl-LDH@GC800/GCE has a fast charge transfer capability and excellent redox capacity, which is attributed to the synergistic effect of Ni bimetals reduced from NiAl-LDH by high-temperature calcination with porous carbon [48].

To explore the electrocatalytic effect of different modified electrodes on TBHQ and its adsorption degree, the modified electrodes were analyzed by differential pulse voltammetry (DPV) (Figure 3C). The response oxidation currents were small, with 0.11 μA and 0.14 μA for NiAl-LDH and NiAl-LDH@GC, respectively. NiAl-LDH@GC800 had the largest oxidation peak current, 20.6 μA, while the oxidation peak currents of the control products NiAl-LDH800 and GC800, which were calcined separately, were almost 0. This indicates that the addition of NiAl-LDH during the GC calcination process improves the electrocatalytic ability of the material greatly such that its modified electrode has better redox properties.

According to the Randles–Sevcik equation [49]:

(1) $$i_{\mathrm{pa}}=2.69\times 10^{5}n^{\frac{3}{2}}D^{\frac{1}{2}}A{C\upsilon }^{\frac{1}{2}}$$

The Randles–Sevcik equation takes into account a variety of parameters and is an important tool for calculating the active surface area of modified electrodes (n = 2, D = 7.6 × 10^−6^ cm^2^·s^−1^, C = 0.005 M). Figure S3A–H shows the electrochemical behavior of the three synthesized materials at different sweep speeds, as observed on the electrochemical workstation. Deriving the linear relationship between i_pa_ and υ^1/2^ by applying the Equation (1), the active surface areas of NiAl-LDH/GCE, NiAl-LDH@GC/GCE, GCE, and NiAl-LDH@GC800/GCE were calculated to be 1.254, 0.8919, 5.173, and 6.068 (mm^2^), respectively. The calculations show that the well-designed NiAl-LDH@GC800/GCE has the largest electrode active surface area, which has more active sites and a higher electron transfer rate. Therefore, NiAl-LDH@GC800/GCE shows higher sensitivity and better detection performance in detecting TBHQ.

### 3.3. Optimization of Experimental Conditions

Electrode drop-coating volume optimization: to explore the influence of the solution volume of the material drop-coated on the electrode on the detection effect, different volumes (2, 4, 6, 8, 10 μL) of NiAl-LDH@GC-800 solution were drop-coated on five glassy carbon electrodes, respectively, and 6 μL was the optimal amount of the drop-coating via the comparison of the detection effect (Figure S4A).

Temperature optimization: the calcination temperature of the material NiAl-LDH@GC was optimized, and NiAl-LDH@GC-700, NiAl-LDH@GC-800, and NiAl-LDH@GC-900 were prepared by taking the appropriate amount of NiAl-LDH@GC at temperatures of 700 °C, 800 °C, and 900 °C, respectively. After the comparison of the tested effects, 800 °C was the optimal calcination temperature for the materials (Figure 3C).

pH optimization: In Figure S4B, it is shown that both oxidation and reduction peak currents increase with the pH change from 5.5 to 7 and decrease with the pH increase from 7 to 8.5. Therefore, 0.1 M PBS at pH 7 was selected as the environment for subsequent electrochemical testing.

Enrichment potential and enrichment time: as shown in Figure S4C, the redox peak current increased with the accumulation time of the TBHQ from 30 s to 180 s. Continuing to push the time to 360 s, the redox peak current changed very little, indicating that the adsorption of TBHQ by NiAl-LDH@GC-800 had reached equilibrium. Therefore, 180 s was selected as the optimal enrichment time. Similarly, we explored the relationship between the enrichment potential and redox peak current at an enrichment time of 180 s (Figure S3D). Based on the results of the electrochemical test experiments, a potential of 0.1 V and a time of 180 s were determined as the set-up test conditions for the workstation.

### 3.4. pH Effect on the Electrochemical Behavior of TBHQ in NiAl-LDH@GC-800/GCE

**Figure 4:**
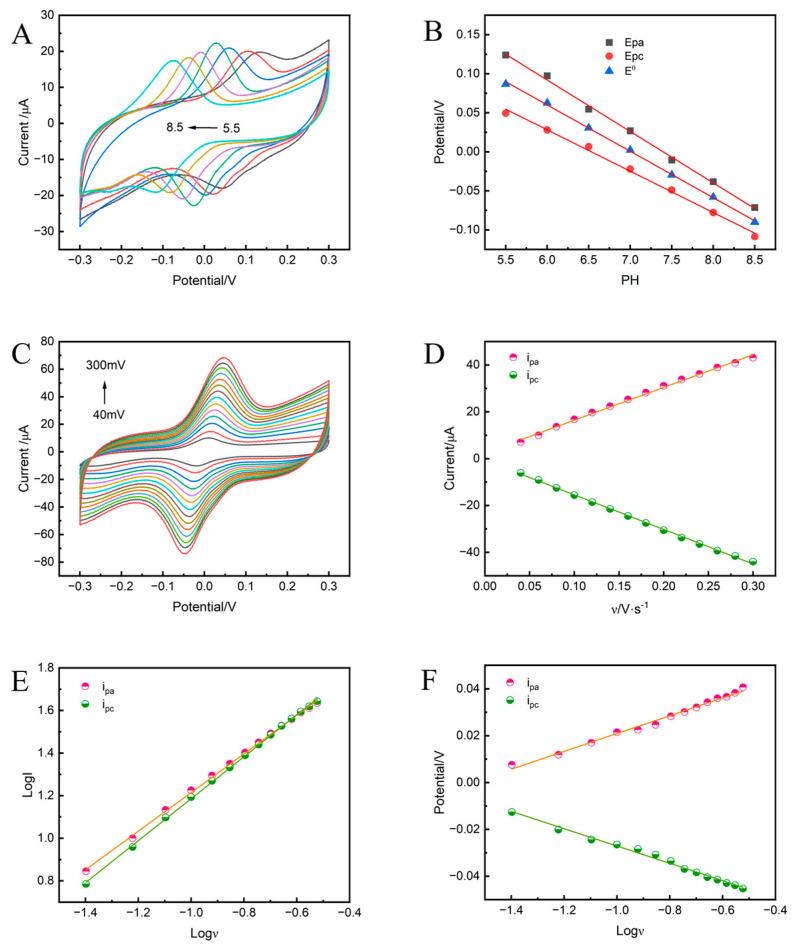
(**A**) CVs of TBHQ on NiAl-LDH @ GC-800/GCE at different pH values in 0.1 M PBS containing 5 × 10^−6^ M TBHQ. (**B**) The dependence of E_pa_ and E_pc_ on pH. (**C**) CV overlay of different scan rates of NiAl-LDH@GC-800/GCE. (**D**) Dependence of the oxidation and reduction peak current on the scan rate. (**E**) Log relationship between i_pa_ and the scan rate. (**F**) Plot of the log of the scan rate versus the potential.

As shown in Figure 4A,B, the redox potential of TBHQ shifted towards negative potential with the increase in the pH of the electrolyte. Three sets of potentials (E_pa_, E_pc_, E^θ^) were calculated to be linearly related to pH:

$$E_{\mathrm{pa}}\left(V\right)=-0.0659\mathrm{pH}+0.4873\left(R^{2}=0.9978\right)\;E_{\mathrm{pc}}\left(V\right)=-0.0529\mathrm{pH}+0.3457\left(R^{2}=0.9957\right)\;E^{\theta }\left(V\right)=-0.0594\mathrm{pH}+0.4165\left(R^{2}=0.9991\right)$$

According to the Nernst equation [50],

(2) $$\frac{d_{Ep}}{d_{pH}}=\frac{2.303mRT}{nF}$$

The application of the equation yields m/n = 0.98, indicating that electron and proton transfers occur essentially simultaneously during the redox process of TBHQ on NiAl-LDH@GC-800/GCE. The slope in the linear fitting equation for the standard potential is 59.4 mV·pH^−1^, which is very much in line with the theoretical value (59 mV·pH^−1^), proving that the redox process of TBHQ at the electrode is an isoelectronic–isoprotonic transfer process [51].

### 3.5. Effect of Scan Rate on the Electrochemical Behavior of TBHQ in NiAl-LDH@GC-800/GCE

We explored the effect of the scan rate on the oxidation and reduction peaks of TBHQ in 0.1 M PBS (Figure 4C–F). In the optimized reaction environment, the redox peak current increased linearly with an increasing scan rate from 0.04 to 0.3 V·s^−1^ (Figure 4C). The calibration equations for the oxidation (i_pa_) and reduction (i_pc_) peaks versus the scan rate ν (V·s^−1^) can be expressed as follows:

$$i_{\mathrm{pa}}\left(\mu A\right)=0.1398\;\nu +2.4479\;{(R}^{2}=0.9967)\;i_{\mathrm{pc}}\left(\mu A\right)=-0.1476\;\nu +0.6945\;(R^{2}=0.9988)$$

The linear dependence of the peak current on scan rate is the characteristic of the oxidation and reduction of the substance adsorbed at the electrode surface. At the same time, we can deduce the mechanism controlling the reaction from the logarithmic relationship that exists between the redox peak and the scan rate [52]. When the slopes of the two are close to 1, it means that the process is controlled by adsorption. Figure 4E demonstrates the excellent linear relationship between the two:

$$Logi_{pa}=0.9078log\upsilon +2.1223(R^{2}=0.9989)\;Logi_{pc}=0.9856log\upsilon +2.1713{(R}^{2}=0.9994)$$

The slopes of the peak redox currents versus the logarithm of the scanning speed are all very close to 1. These results indicate that the redox reaction of TBHQ is an adsorption-controlled process on NiAl-LDH@GC-800/GCE. In addition, based on the CV data, it can be seen that the potential shifts as the scanning speed increases. The following linear relationship exists between the potential and the scanning speed:

$$E_{\mathrm{pa}}\left(V\right)=0.0381\log\upsilon +0.0591{(R}^{2}=0.9904)\;E_{\mathrm{pc}}\left(V\right)=-0.0377\log\upsilon +0.3971(R^{2}=0.9915)$$

According to the Laviron equation [53],

(3) $$E_{pa}\left(V\right)=E_{0}+\left(\frac{2.303RT}{\alpha n_{\alpha }F}\right)\log\left(\frac{RTk_{0}}{\alpha n_{\alpha }F}\right)+\left(\frac{2.303RT}{\alpha n_{\alpha }F}\right)log\upsilon$$

Applying the above equation, we can determine that the value of “*n*_α_” is 2.16, which is approximately equal to 2. These values indicate that the TBHQ redox mechanism at the electrode involves the transfer of two electrons. The two-electron transfer process of TBHQ is drawn in Figure S2. 

### 3.6. Detection Performance of TBHQ at NiAl-LDH@GC-800/GCE

**Figure 5:**
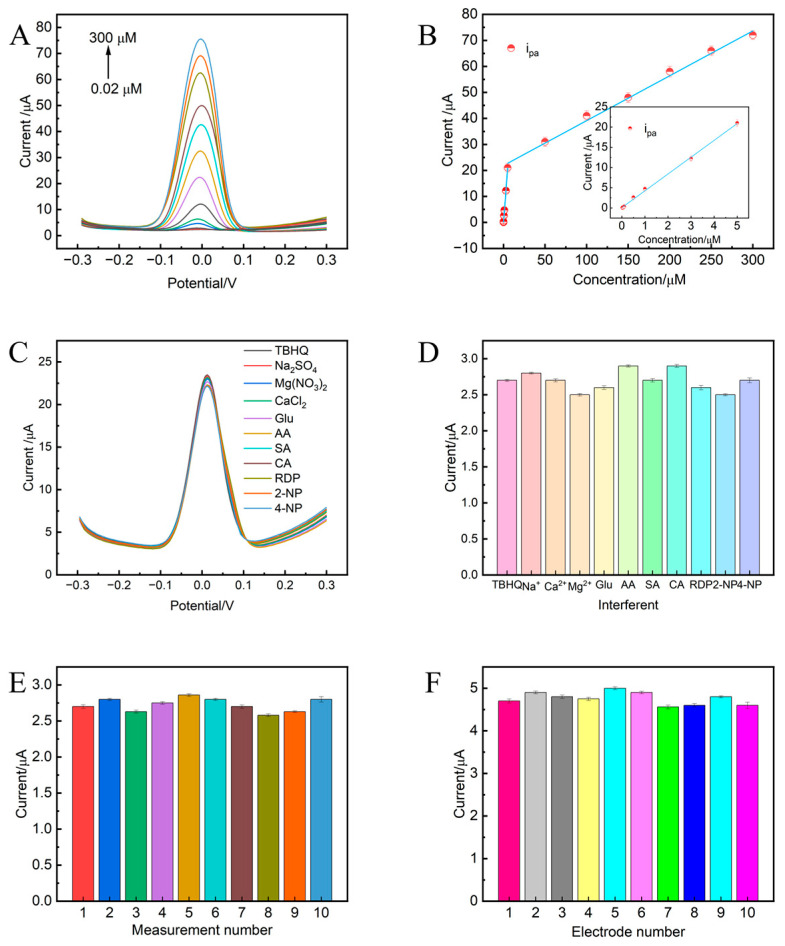
(**A**) DPV responses with different TBHQ concentrations. (**B**) Plot of oxidation peak current value versus TBHQ concentration. (**C**) The DPV curves of TBHQ (5 μM) in PBS (0.1 M) solution containing different interfering substances. (**D**) Demonstration of anti-interference capability (0.5 μM TBHQ). (**E**) Demonstration of the stability of the same modified electrode (10 tests). (**F**) Demonstration of the reproducibility of differently modified electrodes (10 electrodes).

Differential pulse voltammetry (DPV) was used to explore the response behavior of the NiAl-LDH@GC-800 modified electrode regarding different concentrations of TBHQ under the optimal conditions determined (Figure 5A,B). The DPV curves at low concentrations are shown in Supplementary Figure S5. The peak oxidation of DPV showed excellent linearity between 2 × 10^−8^ and 3 × 10^−4^ M TBHQ, and the lowest detected concentration of the composite (LOD = 8.2 × 10^−9^ M) was calculated by applying the formula (D = 3 N/S). N is equal to the relative standard deviation of the response current for the lowest concentration. S is the sensitivity, and the data for S is the slope of the concentration linearity. The excellent linear relationship is expressed as follows:

$$i_{pa}\left(µA\right)=4.1742C_{TBHQ}\left(µM\right)+0.132\left(2\times 10^{-8}M\leq C_{TBHQ}\leq 5\times 10^{-6}M,R^{2}=0.9984\right)\;i_{pa}\left(µA\right)=0.1731C_{TBHQ}\left(µM\right)+22.05\left(5\times 10^{-6}M\leq C_{TBHQ}\leq 3\times 10^{-4}M,R^{2}=0.9939\right)$$

Information on nine TBHQ sensors reported in recent years is listed in Table S2 to evaluate the capability of the sensor proposed in this work. In comparison, our designed porous carbon sensor has a satisfactory linear detection range and detection limit.

### 3.7. The Anti-Interference Ability, Stability, and Reproducibility of the Sensor

To investigate the anti-interference ability of NiAl-LDH@GC-800/GCE, a dozen interfering substances were added in PBS containing 5 μM TBHQ in certain multiples while testing with DPV. The oxidation peak current values changed within 3% when inorganic ions (500 µM Na_2_SO_4_, Mg (NO_3_)_2_, and CaCl_2_), organic acid (50 µM glucose, ascorbic acid, salicylic acid, and citric acid), and molecular analogs (2 µM resorcinol, o-nitrophenol, and p-nitrophenol) were added, indicating that the sensor’s detection of TBHQ is not interfered with by these substances (Figure 5C). Figure S6 serves as a complementary comparison illustrating that none of the DPV curves for the interfering substances had response current peaks in the PBS without TBHQ. In addition, NiAl-LDH@GC-800/GCE showed good anti-interference ability at low TBHQ (0.5 μM) concentrations (Figure 5D and Figure S6B). The stability of the modified electrode continued to be tested under optimal conditions. Ten DPV tests of 0.5 μM TBHQ at NiAl-LDH@GC-800/GCE were carried out (Figure 5E and Figure S6C). The calculated relative standard deviation (RSD) was 2.1%, which indicates that the modified electrode has good stability. The morphology of the material after ten tests was observed by SEM, and it can be seen that the porous structure still exists, indicating the good stability of the material (Figure S7). To evaluate the reproducibility of NiAl-LDH@GC-800 at different electrodes, we analyzed the effect of ten modified electrodes on the detection of 1 μM TBHQ (Figure 5F and Figure S6D). The calculated RSD was 2.9%, which indicated the satisfactory reproducibility of NiAl-LDH@GC-800/GCE for the detection of TBHQ. Finally, we performed electrochemical tests in PBS under an oxygen atmosphere. At three different concentrations of TBHQ, the oxidation peak current in the oxygen atmosphere was slightly smaller than that in the nitrogen atmosphere (Figure S8). This may be because a very small amount of TBHQ reacted with oxygen during the test. The errors were within acceptable limits, which demonstrates the usefulness of the sensor in a practical operating environment.

### 3.8. The Real Sample Analysis

In total, 100 μL of the test sample was added in 20 mL of 0.1 M PBS for electrochemical testing, and then different gradient concentrations of TBHQ were added sequentially. The DPV results were calculated by the calibration equation of the DPV results recorded in Table S3.

**Figure 6:**
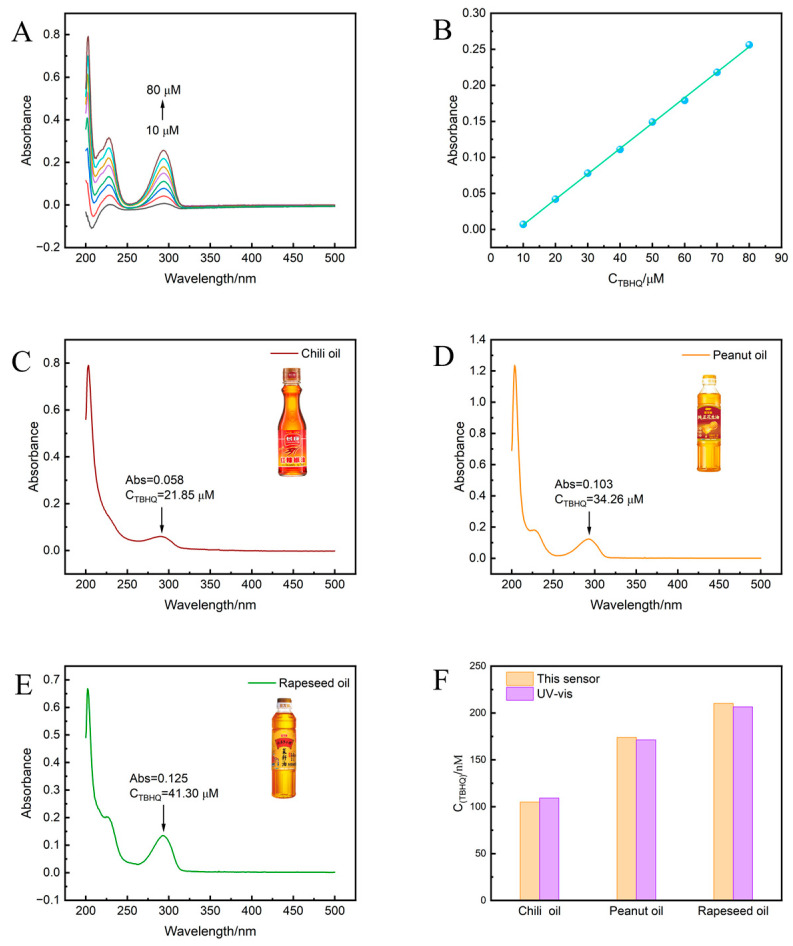
(**A**) UV–vis spectra of TBHQ standard solution (10–80 μM). (**B**) The linearity of absorbance versus TBHQ concentration. UV–vis spectra of (**C**) Chili oil, (**D**) Peanut oil, and (**E**) Rapeseed oil. (**F**) Comparison of TBHQ content in real samples measured by UV and sensor methods.

In addition, we verified the detection accuracy of NiAl-LDH@GC-800/GCE using UV-visible spectrophotometry. We prepared a series of TBHQ standard solutions (10–80 μM) using ethanol as the solvent and established a TBHQ standard curve using UV-visible spectrophotometry (Figure 6A,B). The results show that there is a linear correlation between the concentration of TBHQ and the absorption peak at a wavelength of about 292 nM in the UV-visible spectrophotometric method, which is very close to 1, and the formula is as follows:

$$Abs=0.0035C_{TBHQ}+0.0288(10\;\mu M\leq C_{CGA}\leq 80\;\mu M\;\;R^{2}=0.9994)$$

The absorption peaks of the three diluted actual sample solutions at specific wavelengths were determined using UV-visible spectrophotometry (Figure 6C–E). By comparison of the results obtained by the developed electrochemical method with the results of the spectrophotometric determination, it can be found that the sensor consisting of NiAl-LDH@GC-800/GCE has a good test accuracy (Figure 6F). This shows that the NiAl-LDH@GC-800/GCE prepared by us has high accuracy for the detection of TBHQ and can be considered for the detection of TBHQ content in real food samples.

Based on the above results, it is concluded that the sensor is made of low-priced and easily available raw materials, and it has good linearity and stability. The sensors have good anti-interference ability and significant potential for real sample detection. However, the high temperature of 800 °C and the multiple material synthesis steps are disadvantages for the commercialization of the sensor.

## 4. Conclusions

In this paper, a novel electrochemical sensor based on porous metal carbon materials derived from GC and NiAl-LDH nanocomposites was proposed and successfully used for TBHQ detection in real food samples. The NiAl-LDH@GC-800 material obtained from pyrolysis has a porous structure and exposes a larger specific surface area than the precursor, which promotes the adsorption of TBHQ on the sensor and significantly enhances the electrochemical signal. The synergistic effect of porous carbon and Ni metal reduced from NiAl-LDH by high-temperature calcination accelerated the electron transfer rate and improved the sensitivity of the sensors. Under optimized conditions, the sensor showed good sensing ability in TBHQ detection, with a linear range of 0.02–30 μM and an LOD of 8.2 nM. The present work proved the excellent performances of metallic porous carbon materials in constructing an electrochemical sensor and also provides an alternative method for the analysis and determination of antioxidants in food.

## Data Availability

The original contributions presented in the study are included in the article/Supplementary Materials, further inquiries can be directed to the corresponding author.

## Supplementary Materials

The following supporting information can be downloaded at: https://www.mdpi.com/article/10.3390/foods13213431/s1, Figure S1: (A) FTIR spectra of GC, NiAl-LDH, NiAl-LDH@GC, and NiAl-LDH@GC800. (B) Zeta data of GC NiAl-LDH, NiAl-LDH@GC, and NiAl-LDH@GC800; Figure S2: (A) Possible redox processes of TBHQ on the NiAl-LDH@GC800 sensor, Figure S3: CVs of NiAl-LDH/GCE (A,B), NiAl-LDH@GC/GCE (C,D), and NiAl-LDH @ GC800/GCE (E,F) in a 5 mM [Fe(CN)6]^3−/4−^ probe containing 0.1 M KCl.; Figure S4: (A) Peak oxidation currents of NiAl-LDH@GC800/GCE in 0.1 M PBS (pH = 7) containing 5 × 10^−6^ M TBHQ at different droplet amounts. (B) The oxidation peak currents of 0.1 M PBS with different pH values. (C) The influence of enrichment time on oxidation peak current. (D) The influence of enrichment potential on oxidation peak current; Figure S5: DPV responses of different TBHQ concentrations (0.02 µM, 0.05 µM, 0.1 µM, 0.5 µM,1 µM, 3 µM, 5 µM, 50 µM, 100 µM, 150 µM, 200 µM, 250 µM, 300 µM); Figure S6: (A) DPV curves of different interfering substances in 0.1 M PBS solution (without TBHQ). (B) DPV curves of TBHQ (0.5 μM) in PBS containing different interfering substances. (C) DPV curves of TBHQ (0.5 μM) in PBS (10 tests). (D) DPV curves of TBHQ (1 μM) in PBS (10 electrodes); Figure S7: (A,B) SEM images of NiAl-LDH@GC800 before electrochemical measurements. (C,D) SEM images of NiAl-LDH@GC800 after electrochemical measurements; Figure S8: Comparison of detection in nitrogen and oxygen environments (A) 5 μM TBHQ. (B) 1 μM TBHQ. (C) 0.5 μM TBHQ. (D) Oxidation peak current comparison; Table S1: Comparative evaluation of the performance of NiAl-LDH@GC800/GCE with other modified electrodes in TBHQ detection; Table S2: Detection of TBHQ in Different Edible Oils (n = 3); Table S3: Detection of TBHQ in Different Edible Oils (n = 3).
