# Supplementary material for: Detection of Tert-Butylhydroquinone in Edible Oils Using an Electrochemical Sensor Based on a Nickel-Aluminum Layered Double Hydroxide@Carbon Spheres-Derived Carbon Composite
Source: Foods. 2024 Oct 28;13(21):3431. doi: 10.3390/foods13213431 (PMC11545052; doi:10.3390/foods13213431)
Supplement: Supplementary file 1 [file foods-13-03431-s001.zip › foods-3188887-supplementary.pdf]

Supplementary Material

Detection of Tert-Butylhydroquinone in Edible Oils Using an Electrochemical Sensor Based on a Nickel-Aluminum Layered Double Hydroxide@Carbon Spheres-Derived Carbon Composite

Jin Zhang [1,2], Jingrong Chen [3], Jiejun Li [3] and Yixi Xie [1,3,*]

[1] School of Materials and Chemical Engineering, Hunan City University, Yiyang 413000, China; tccdzdc@163.com

[2] Key Laboratory of Low Carbon and Environmental Functional Materials of College of Hunan Province, Yiyang 413000, China

[3] Hunan Provincial University Key Laboratory for Environmental and Ecological Health, Xiangtan University, Xiangtan 411105, China

* Correspondence: xieyixige@xtu.edu.cn; Tel.: +86-731-58292259

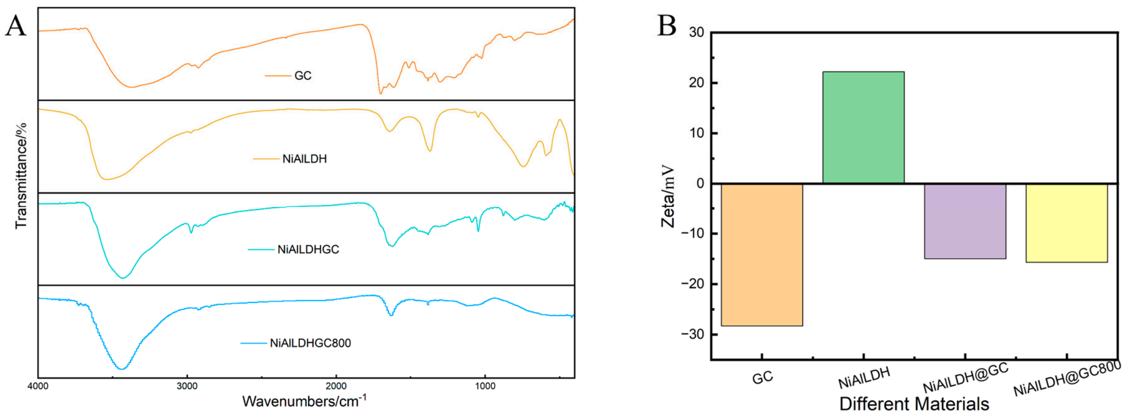

Figure S1. (A) FTIR spectra of GC, NiAl-LDH, NiAl-LDH@GC, and NiAl-LDH@GC800. (B) Zeta data of GC NiAl-LDH, NiAl-LDH@GC , and NiAl-LDH@GC800.

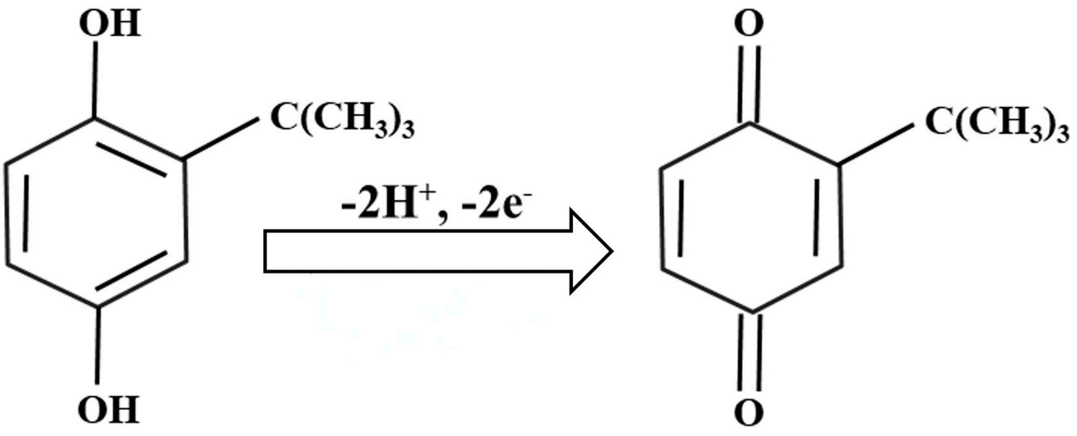

Figure S2. (A) Possible redox processes of TBHQ on the NiAl-LDH@GC800 sensor.

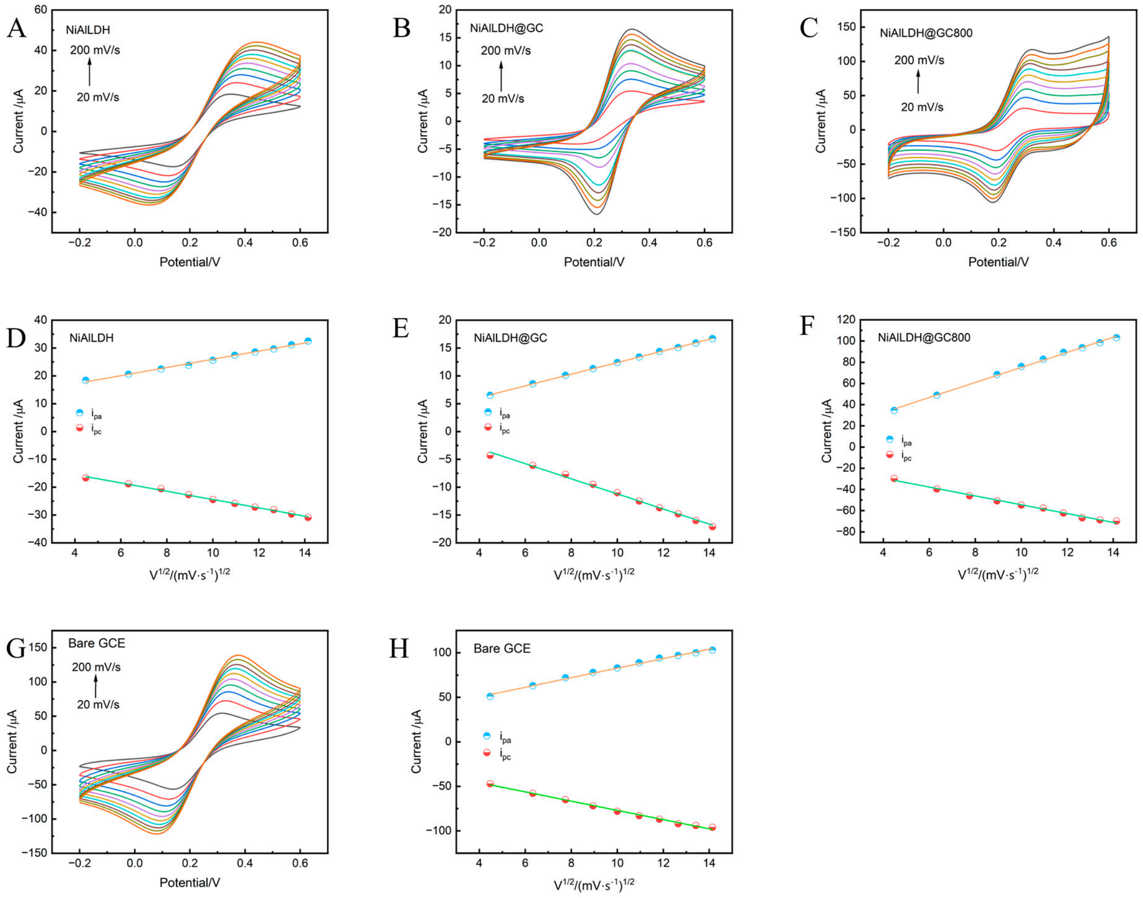

Figure S3. CVs of NiAl-LDH/GCE(A), NiAl-LDH@GC/GCE(B), NiAl-LDH @ GC800/GCE (C) and GCE (G) in a 5 mM $[Fe(CN)_6]^{3-/4-}$ probe containing 0.1 M KCl at different scan rates. (D, E, F, H)Oxidation peak current versus scan rate for each modified electrode.

.

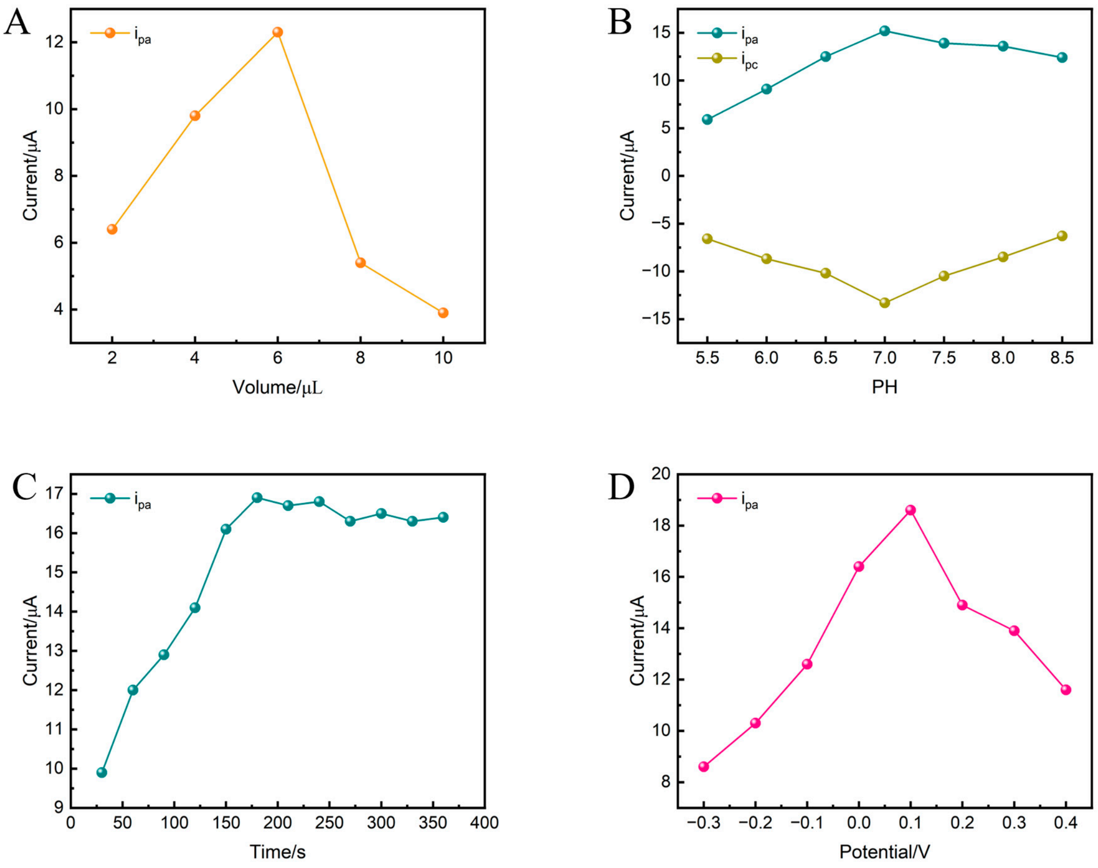

Figure S4. (A) Peak oxidation currents of NiAl-LDH@GC800/GCE in 0.1 M PBS (pH=7) containing 5×10^{-6} M TBHQ at different droplet amounts. (B) the oxidation peak currents of 0.1 M PBS with different pH values. (C) the influence of enrichment time on oxidation peak current. (D) the influence of enrichment potential on oxidation peak current.

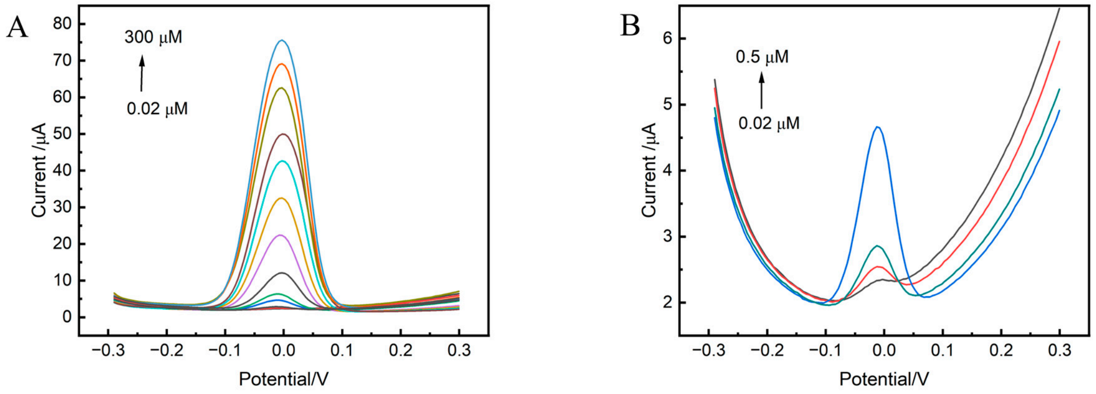

Figure S5. DPV responses of different TBHQ concentrations (0.02 μM, 0.05 μM, 0.1 μM, 0.5 μM,1 μM, 3 μM, 5 μM, 50 μM, 100 μM, 150 μM, 200 μM, 250 μM, 300 μM).

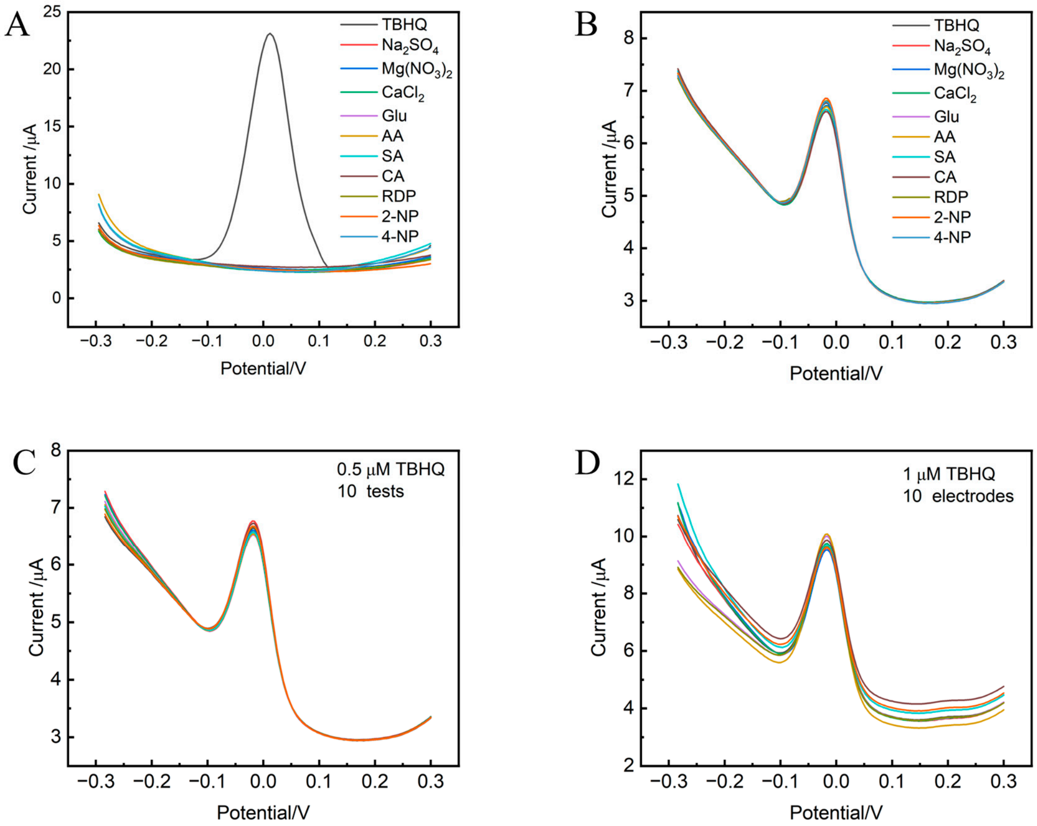

Figure S6. (A) DPV curves of different interfering substances in 0.1 M PBS solution (without TBHQ). (B) DPV curves of TBHQ (0.5 μM) in PBS containing different interfering substances. (C) DPV curves of TBHQ (0.5 μM) in PBS (10 tests). (D) DPV curves of TBHQ (1 μM) in PBS (10 electrodes).

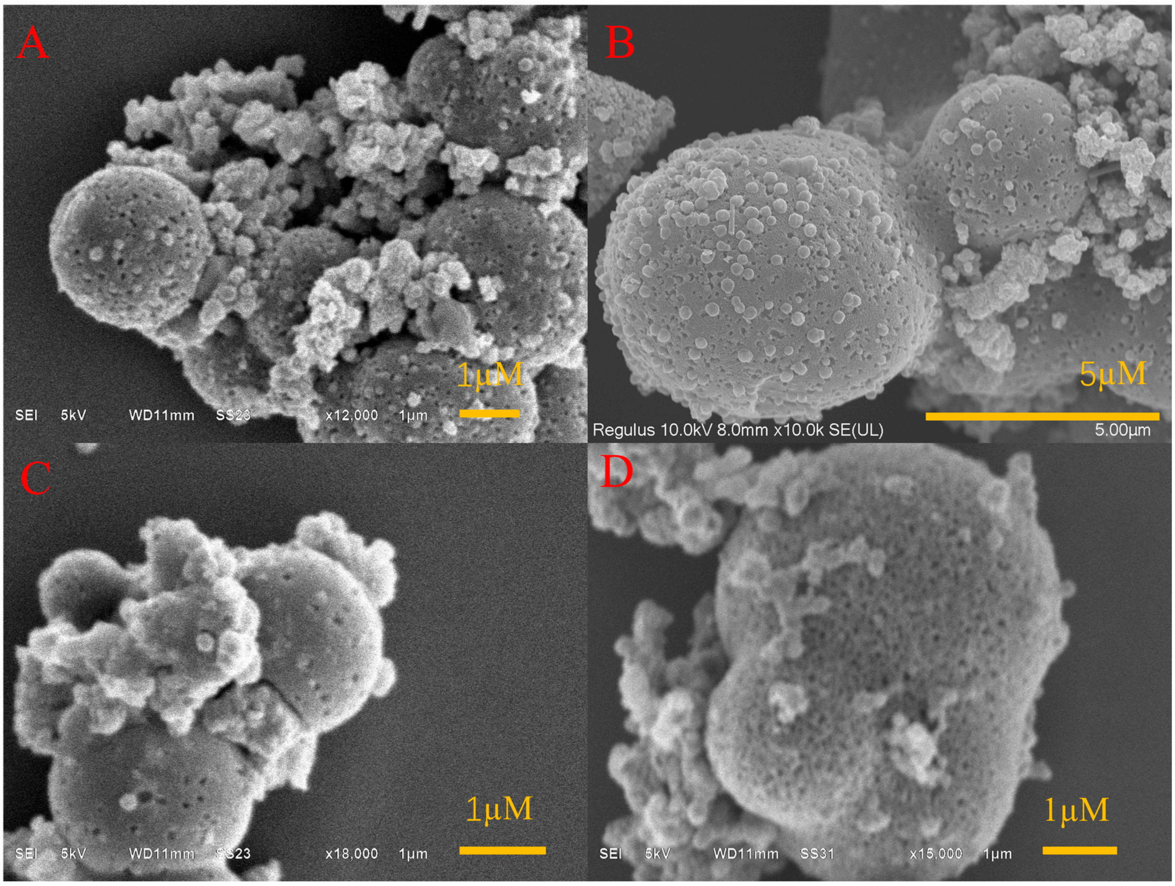

Figure S7. (A-B) SEM images of NiAl-LDH@GC800 before electrochemical measurements. (C-D) SEM images of NiAl-LDH@GC800 after electrochemical measurements.

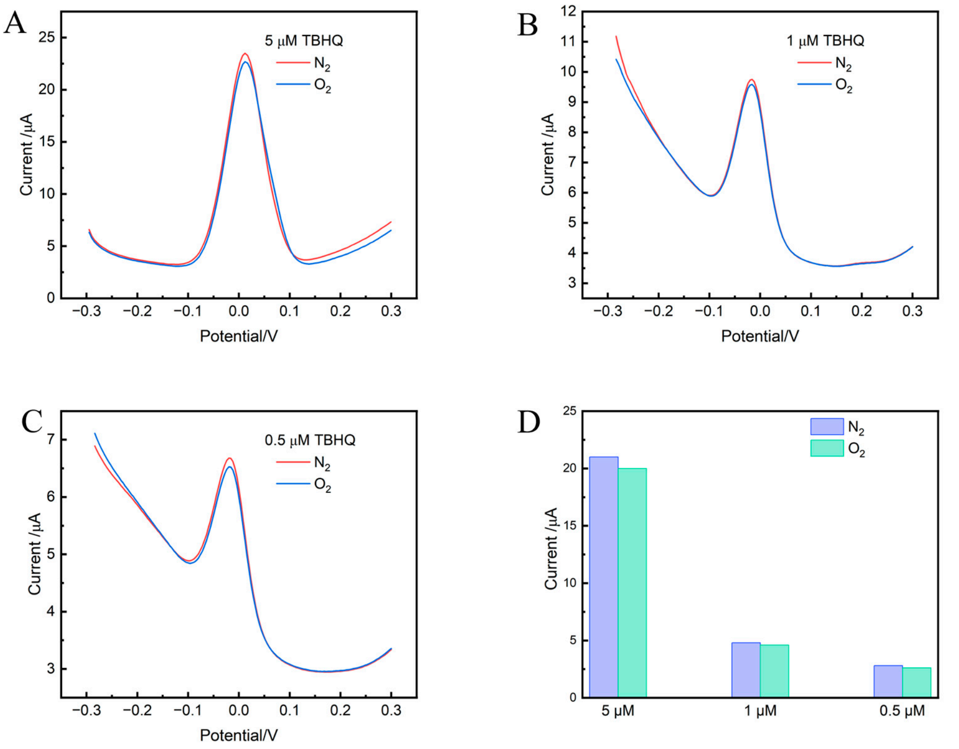

Figure S8. Comparison of detection in nitrogen and oxygen environments (A) 5 μM TBHQ. (B) 1 μM TBHQ. (C) 0.5 μM TBHQ. (D) Oxidation peak current comparison.

Table S1. CV data for modified electrodes in a 5.0 mM $[Fe(CN)_6]^{3-/4-}$.

Modified electrode	I_{pa} (μA)	I_{pc} (μA)	E_{pa} (V)	E_{pc} (V)	ΔE_p (V)
NiAl-LDH	25.63	-23.71	0.372	0.091	0.281
NiAl-LDH@GC	12.51	-12.19	0.313	0.186	0.127
NiAl-LDH@GC800	76.73	-75.84	0.304	0.191	0.113
GCE	58.71	-53.16	0.276	0.151	0.125

Table S2. Comparative evaluation of the performance of NiAl-LDH@GC800/GCE with other modified electrodes in TBHQ detection.

Modified electrode	Detection technique	LOD (μM)	Linear range (μM)	References
MIP-MWCNT/GCE	DPV	0.85	2.84-150	(Santos Moretti et al., 2016)
MIP/MoS_2/EACC	DPV	0.00072	0.001-120	(Chi et al., 2024)
MIP/AuNPs/EGP	DPV	0.07	0.08-100	(Fan et al., 2018)
$FeNi_3$/rGO/$HMPF_6$/CPE	SWV	0.01	0.05-900	(Tahernejad-Javazmi et al., 2019)
PVP–CTAB/Au–PVP–Gr/GCE	DPV	0.009	0.02-0.1, 0.1-100	(Wang et al., 2016)
ZnCuMg TMO/β-CD-CB/SPCE	DPV	0.001	0.031-12.56, 12.56-118.8	(Sebastian, et al., 2022)
ZnO TPHS@GO/GCE	SWV	0.137	0.8-65	(Gan et al., 2016)
Co NC/CNT/MB/ GCE	DPV	0.054	0.1-20, 20-100	(Zhang et al., 2024)
NiAl-LDH@GC800/GCE	DPV	0.0082	0.02-5, 5-300	This work

Table S3. Detection of TBHQ in Different Edible Oils (n=3)

sample	recruitment (nM)	Discovery quantity (nM)	recovery rate (%)	RSD (%)	UV-Vis (test)	UV-Vis (dilution)
chili oil	0	105	—	—	21.85 μM	109.2 nM
	40	143.8	97.0	1.7		
	80	188	103.7	2.3		
	120	223	102.5	2.1		
peanut oil	0	174	—	—	34.26 μM	171.3 nM
	50	225.7	103.4	2.6		
	100	276.2	102.2	1.9		
	150	329.6	103.6	2.3		
rap oil	0	210.3	—	—	41.30 μM	206.5 nM
	50	259.4	98.2	2.2		
	100	311.3	101.0	1.5		
	150	363.6	102.2	2.0		

Relative standard deviation (RSD) is as follows:

$RSD = \frac{SD}{\bar{x}} \times 100\% = \frac{\sqrt{\frac{\Sigma_{i=1}^{n}(x_i-\bar{x})^2}{n-1}}}{\bar{x}} \times 100\%$, where SD is the standard deviation and $\bar{x}$ is the mean.
